# Radical resection and reconstruction in patients with adenoid cystic carcinoma in the minor salivary glands of the palate

**DOI:** 10.1186/s13005-022-00312-7

**Published:** 2022-03-16

**Authors:** Wei-liang Chen, Yan Wang, Bin Zhou, Juan-kun Liao, Rui Chen

**Affiliations:** 1 Department of Oral and Maxillofacial Surgery, Sun Yat-sen Memorial Hospital, Sun Yat-sen University, 107 Yan-jiang Road, Guangzhou, 510120 China; 2 Department of Oral and Maxillofacial Surgery, Sun Yat-sen Memorial Hospital, Sun Yat-sen University, Guangzhou, 510120 China; 3 Department of Oral and Maxillofacial Surgery, Sun Yat-sen Memorial Hospital, Sun Yat-sen University, Guangzhou, 510120 China; 4 Department of Oral and Maxillofacial Surgery, Sun Yat-sen Memorial Hospital, Sun Yat-sen University, Guangzhou, 510120 China; 5 Department of Oral and Maxillofacial Surgery, Sun Yat-sen Memorial Hospital, Sun Yat-sen University, Guangzhou, 510120 China

**Keywords:** Adenoid cystic carcinoma, Minor salivary gland, Palate, Flap, Ki-67 expression, Surgical procedures

## Abstract

**Background:**

This study evaluated the clinical outcomes of the patients with adenoid cystic carcinoma (ACC) of the minor salivary glands of the palate.

**Methods:**

Forty-four patients with stage I–II disease and 14 patients with stage III–IV disease underwent radical excision and reconstruction with a facial-submental artery island flap (FSAIF) and titanium mesh plus a free anterolateral thigh flap (ALTF) and radiotherapy respectively. Patients with stage III–IV disease subsequently received cobalt Co 60 adjuvant radiotherapy. Ki-67 expression was determined semiquantitatively in 52 patients with ACC by based on the cytoplasm staining intensity and percentage of positively stained tumor cells.

**Results:**

The median (range) follow-up was 32.9 (14–58) months. Forty-one (71.7%) patients survived without disease recurrence. Nine patients (15.5%) survived with recurrent tumors (four with local recurrence, three with regional recurrence requiring salvage surgery, and two with distant metastasis); among these patients, five had overlapping recurrence. Eight patients (13.8%) died of regional, distant, or multiorgan metastasis (range: 22–42 months). The overall median (95% CI) survival time was 32.5 (25.0–39.5) months, and the median (95% CI) progression-free survival time was 32.9 (28.5–36.9) months. Rates of survival and recurrence differed significantly between patients with low- and high-grade tumors, patients with clinical stage I–II disease and those with stage III–IV disease, patients with and without lymph node metastasis, patients who underwent radical excision with versus without radiotherapy, and patients with low and high Ki-67 expression.

**Conclusion:**

Radical resection and reconstruction with FSAIF is suitable methods for the the treatment of stage I–II ACC of the minor salivary glands of the palate. Stage III–IV tumors require radical resection, reconstruction with titanium mesh and free ALTF, and radiotherapy.

## Introduction

Approximately 41.5% of all salivary gland tumors are minor salivary gland neoplasms, and almost 60% of tumors arising from the minor salivary glands are malignant [1, 2]. The palate is the most common site for intraoral minor salivary gland carcinomas [3, 4], and adenoid cystic carcinoma (ACC) is the most common histologic type [5]. ACC is a malignant tumor composed of ductal cells and abluminal modified myoepithelial basaloid cells showing various microscopic patterns [6]. The growth patterns are categorized as *cribriform*, *tubular*, and *solid*. A mixture of these patterns usually occurs within a single tumor, but the foci of cribriform tumors can usually be found even when another type predominates [7]. Although slow growing, ACC is a life-threatening malignant tumor owing to its high risk of recurrence; of the rate of tumor-related death within 30 years after primary treatment is high [8]. However, little research has been performed on the surgical outcomes of patients with ACC originating from the minor salivary glands of the palate. Here, we evaluated the outcomes of patients with ACC of the minor salivary glands of the palate who underwent radical excision and flap reconstruction.

## Patients and methods

This retrospective observational study was conducted from January 2011 through December 2019 at the Department of Oral and Maxillofacial Surgery, Sun Yat-sen Memorial Hospital, Sun Yat-sen University, Guangzhou, China. The 58 patients with ACC of the minor salivary glands of the palate enrolled in the study had primary tumors. We collected data on age, sex, size of tumor, histologic diagnosis, classifications for tumors, treatment, rates of survival and recurrence. Diagnostic imaging of the primary lesion was performed using three-dimensional computed tomography (3D-CT) and magnetic resonance imaging (MRI). Age groups are classified according to the cut-off age of the “elderly” recommended by the WHO [9]; The histologic diagnosis was confirmed according to the 2017 WHO classifications for salivary gland tumors [10]; The clinical stages were classified according to the American Joint Committee on Cancer’s *Cancer Staging Manual* (8th edition) [11]; The defects of maxilla and midface were classified by Brown classification [12]; All submandibular lymph nodes were checked during FSAIF elevation and details of the surgery were provided by Chen et al. [13]; All intraoperative proximal margin frozen section (FS) specimens were classified as R0 (FS analysis showed negative surgical margins) or R1 (FS analysis showed negative surgical margins after previous identification of positive margins and additional resection). Ki-67 expression was determined semiquantitatively based on the cytoplasm staining intensity and percentage of positively stained tumor cells [14]. The exclusion criteria were cachexia, congestive cardiac failure, severe chronic obstructive pulmonary disease, and/or lack of follow-up data.

### Statistical analyses

Statistical analyses were performed IBM SPSS Statistics (version 22.0; IBM Corp., Armonk, NY, USA). Overall survival (OS) and progression-free survival (PFS) were estimated with Kaplan–Meier curves. The chi-square test was used to analyze the data, as appropriate. A *P*-value of ≤0.05 was considered significant. The Institutional Review Board of Sun Yat-sen University approved this study.

## Results

**Fig. 1:**
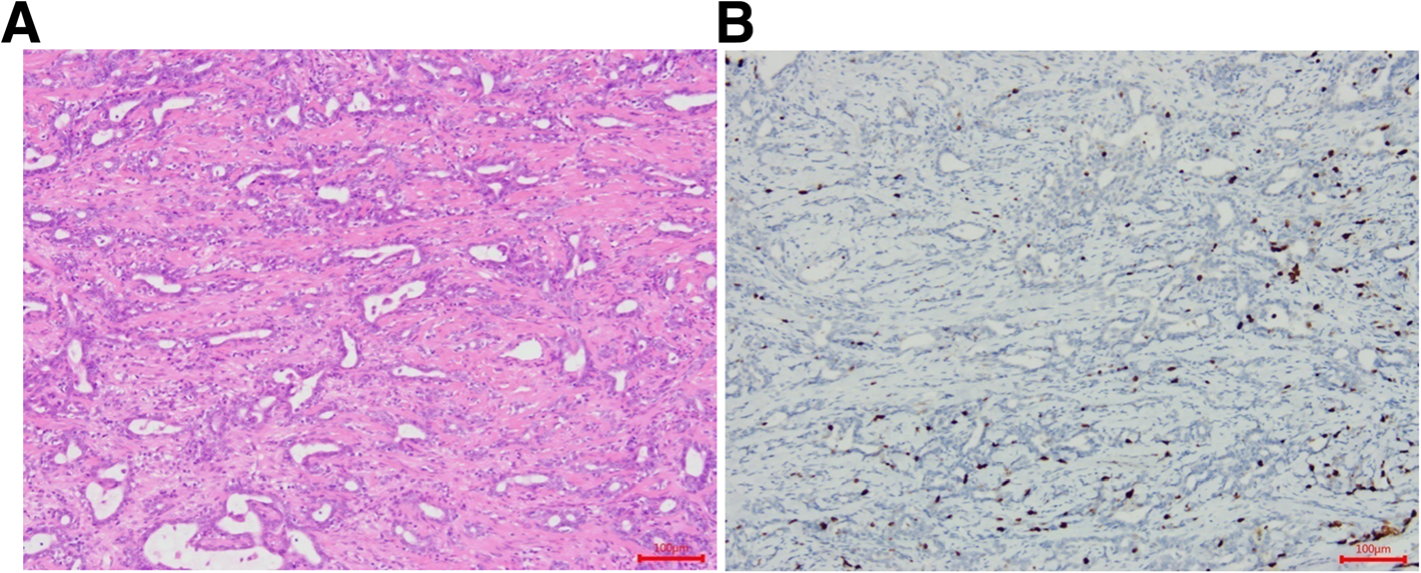
Grade I tumors showing only a tubular and cribriform pattern without solid components. Solid-type adenoid cystic carcinoma (ACC) with elements of the cribriform and tubular types (hematoxylin and eosin staining) (**A**). Immunohistochemical staining for Ki-67 in ACC showing 10% positive cells (**B**)

**Fig. 2:**
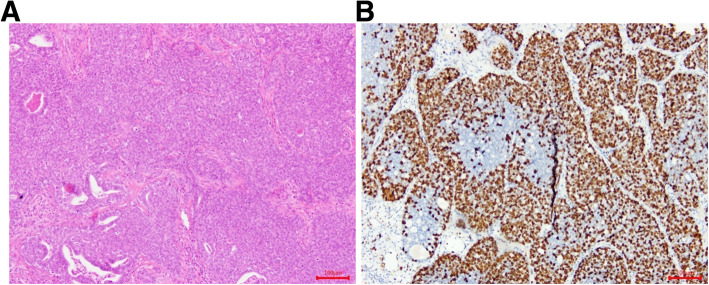
Grade III tumors showing 60% solid components (hematoxylin and eosin staining) (**A**). Immunohistochemical staining for Ki-67 in adenoid cystic carcinoma cells, the staining intensity is strong, showing 60% positive cells (**B**)

A total of 58 patients with ACC of the minor salivary glands of the palate were identified who had flap reconstruction following radical resection at the Department of Oral and Maxillofacial Surgery, Sun Yat-sen Memorial Hospital, Sun Yat-sen University (Guangzhou, Guangdong, China). Of the 58 patients, 28 were male and 30 were female. Patient age ranged from 20 to 80 years (median ± standard deviation, 49.2 ± 9.8 years). We classified patients into ≤60 years (*n* = 47) and > 60 years (*n* = 11) age groups according to the cut-off age for “elderly” recommended by the World Health Organization (WHO) [9]. All patients exhibited slow-growing, painless swelling of the palate that began several months prior. Tumors were classified according to size, i.e., as ≤2 cm (*n* = 9) or > 2 cm (*n* = 49). In all cases, histologic diagnosis was confirmed according to the 2017 WHO classifications for salivary gland tumors [10]. ACC tumors were histopathologically classified as grade I–III. Grade I tumors showed a tubular and cribriform pattern without solid components (*n* = 14) (Fig. 1); grade II tumors were cribriform with < 30% solid components (*n* = 18); and grade III tumors had ≥30% solid components (*n* = 24) (Fig. 2). Tumors with an area of histologic transformation were classified as *transformed* (*n* = 3).

**Fig. 3:**
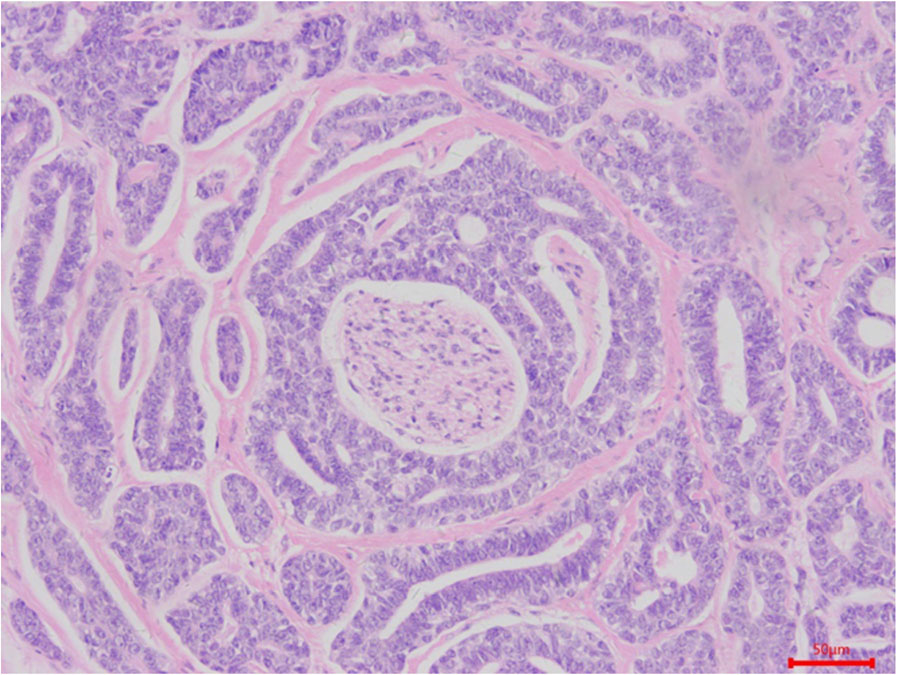
Histologic section showing perineural invasion by ACC cells (hematoxylin and eosin staining)

**Fig. 4:**
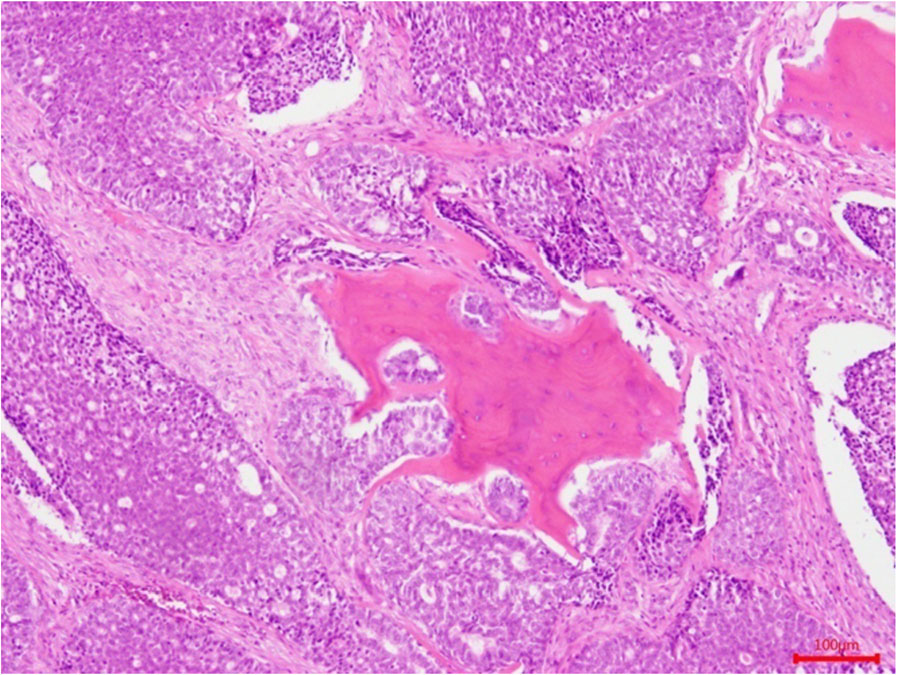
Histologic section showing bone invasion by ACC cells (hematoxylin and eosin staining)

**Fig. 5:**
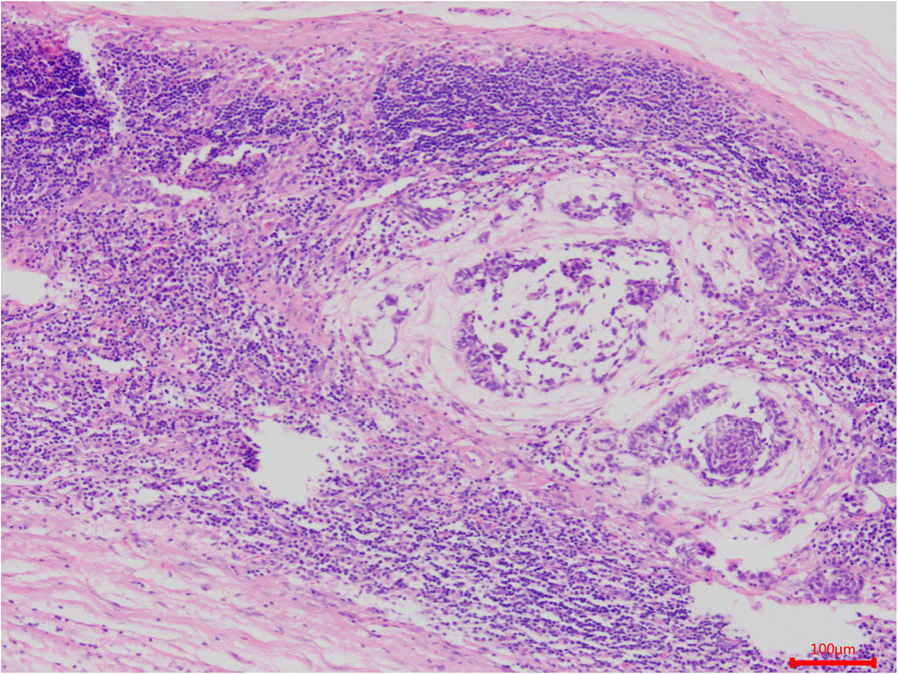
Histologic section showing lymph node metastasis by ACC cells (hematoxylin and eosin staining)

Perineural invasion, bone invasion, and lymph node metastasis—which were defined as the presence of ACC cells in the nerve fiber (Fig. 3), maxillary bone (Fig. 4), and lymph nodes of the neck (Fig. 5) on histological examination—were noted in 48 (82.8%), 52 (89.7%), and 3 (5.2%) patients, respectively. According to the classifications of the American Joint Committee on Cancer’s *Cancer Staging Manual* (8th edition) [11], 9 (15.5%), 35 (60.3%), 11 (19.0%), and 3 patients (5.2%) had clinical stage I–IV disease, respectively.

**Fig. 6:**
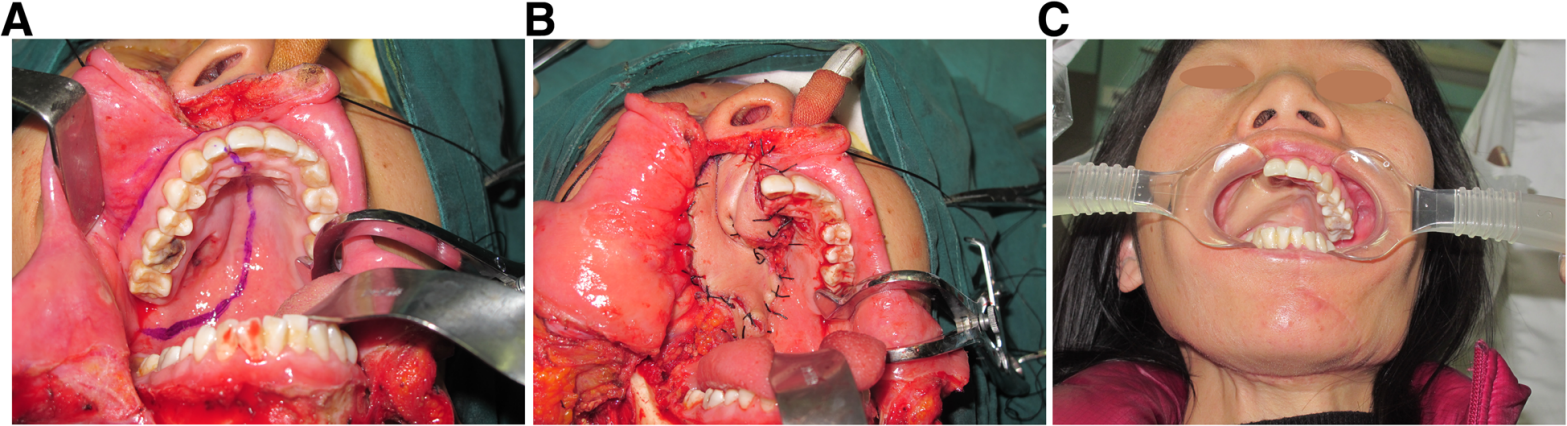
A 31-year-old women with stage II palatal ACC underwent subtotal maxillectomy and ipsilateral selective neck dissection. Incision of the tumor and the defect (**A**). Reconstruction of class II maxillary defects with a facial-submental artery island flap (**B**). The photograph was taken 24 months after surgery (**C**)

**Fig. 7:**
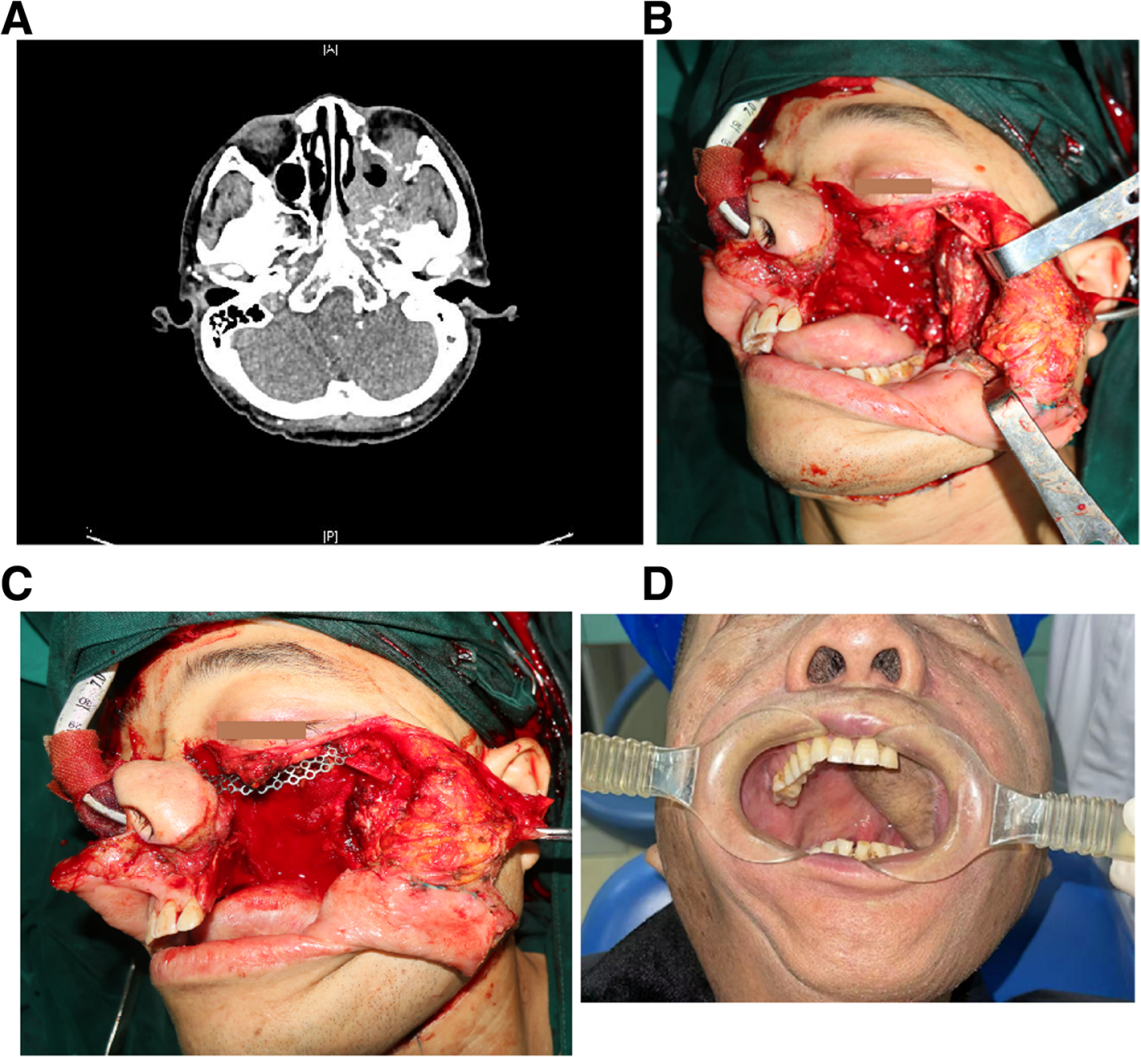
A 51-year-old man with stage IV palatal ACC underwent total maxillectomy and ipsilateral selective neck dissection. Computed tomography scan showing that the tumor had broken through the inferior orbital wall and entered the orbit (**A**). Incision of the tumor and defect (**B**). Reconstruction of the inferior orbital wall and class III maxillary defects with titanium mesh (**C**) and a free anterolateral thigh flap (**D**)

Forty-four patients with stage I-II disease underwent radical excision, including subtotal maxillectomy (intraoral approach) and ipsilateral selective neck dissection. According to the Brown classification for maxillary and midface defects [12], 44 patients had class II maxillary defects (requiring subtotal maxillectomy not involving the orbital floor or adnexa) that were reconstructed with a facial-submental artery island flap (FSAIF) based on the distal facial pedicle (Fig. 6). Fourteen patients with stage III and IV disease underwent radical excision, including total maxillectomy (via the Weber–Ferguson approach) with preservation of the orbital contents, and ipsilateral selective neck dissection. Class III maxillary defects (requiring total maxillectomy and loss of orbital support) were reconstructed with titanium mesh and a free anterolateral thigh flap (ALTF) (Fig. 7).

All submandibular lymph nodes were checked during flap elevation and confirmed as pathologically negative before we harvested the FSAIF. Details of the surgery were provided in a 2008 report [13]. All intraoperative proximal margin frozen section (FS) specimens were classified as R0 (FS analysis showed negative surgical margins) or R1 (FS analysis showed negative surgical margins after previous identification of positive margins and additional resection). Resection status was R0 in 56 patients (96.6%) and R1 in 2 (3.4%). Fourteen patients with stage III and IV were treated with surgical excision followed by cobalt Co 60 adjuvant radiotherapy for the primary tumor site, and the interval between surgery and radiotherapy was 30 days. In total, 60 Gy was administered over 30 days with a conventional dose of 2 Gy fractions/day.

We used immunohistochemistry to analyze Ki-67 expression in paraffin-embedded specimens obtained from 52 patients with ACC. Ki-67 expression was determined semiquantitatively based on the cytoplasm staining intensity and percentage of positively stained tumor cells [14]. Staining intensity was scored as 0, indicating no staining or weak staining; 1, moderate staining; or 2, strong staining. The percentage of immunoreactive tumor cells was scored as 0, representing < 10% positivity (Fig. 1B); 1, 10–50%; or 2, > 50% (Fig. 2B). The overall Ki-67 expression score thus ranged from 0 to 4, i.e., the sum of the points for the percentage of positively stained cells and staining intensity. For statistical analysis, patients were divided into two groups: those with scores of 0–2 were considered to have low Ki-67 expression, and those with scores of 3–4 were considered to have high expression [8]. Ki-67 expression was low in 40 patients (76.9%) and high in 12 patients (23.1%). However, high Ki-67 expression was identified in 3 of 28 patients (10.7%) with low-grade tumors and 9 of 24 patients (37.5%) with high-grade tumors. Table 1 summarizes the demographic and clinical characteristics of the patients with palatal ACC.

**Table 1:** Demographic characteristics, clinical characteristics and outcomes of 58 patients with palatal adenoid cystic carcinoma

Parameter	No. Of cases (%)	Survival without disease (%)	Survival with recurrence (%)	Death (%)	***P***-value
**Sex**					**0.683**
Male	28 (48.3)	19/28 (67.9)	4/28 (14.2)	5/28 (17.9)	
Female	30 (51.7)	22/30 (73.3)	5/30 (16.7)	3/30 (10.0)	
**Age (y)**					**0.665**
≤ 60 years	47 (81.0)	32/47 (68.1)	8/47 (17.0)	7/47 (14.9)	
> 60 years	11 (19.0)	9/11 (81.8)	1/11 (9.1)	1/11 (9.1)	
**Tumor size (cm)**					**0.109**
≤ 2 cm	9 (15.5)	9/9 (100.0)	0/9 (00.0)	0/9 (0.0)	
> 2 cm	49 (84.5)	32/49 (65.3)	9/49 (18.4)	8/49 (16.3)	
**Histopathologic grade**					**0.0001**
Low grade (I + II)	(14 + 18) (55.2)	30/32 (90.6)	0/32 (0.00)	2/32 (6.3)	
High grade (III + transformed)	(23 + 3) (44.8)	11/26 (42.3)	9/26 (34.6)	6/26 (23.1)	
**Perineural invasion**					**0.281**
Absent	10 (17.2)	9/10 (90.0)	1/10 (10.0)	0/10 (0.0)	
Present	48 (82.8)	32/48 (66.6)	8/48 (16.7)	8/48 (16.7)	
**Bone invasion**					**0.250**
Absent	6 (10.3)	6/6 (100.0)	0/6 (00.0)	0/6 (100.0)	
Present	52 (89.7)	35/52 (67.3)	9/52 (17.3)	8/52 (15.4)	
**Lymph node metastasis**					**0.015**
Absent	55 (94.8)	41/55 (74.6)	7/55 (12.7)	7/55 (12.7)	
Present	3 (5.2)	0/3 (0.0)	2/3 (66.7)	1/3 (33.3)	
**TNM stage**					**0.0001**
Early (I + II)	(9 + 35) (75.9)	39/44 (88.7)	2/44 (4.5)	3 /44 (6.8)	
Advanced (III + IV)	(11 + 3) (24.1)	2/14 (14.3)	7/14 (50.0)	5/14 (35.7)	
**Treatment**					**0.004**
Radical excision	44 (75.9)	36/44 (81.8)	4/44 (9.1)	4/44 (9.1)	
Radical excision with radiotherapy	14 (24.1)	5/14 (35.7)	5/14 (35.7)	4/14 (28.6)	
**Surgical margin**					**0.081**
R0	56 (96.6)	41/56 (73.2)	8/56	7/56	
R1	2 (3.4)	0/2 (0.0)	(14.3) 1/2 (50.0)	(12.5) 1/2 (50.0)	
**Ki-67 expression**^**a**^					**0.003**
Low	40 (76.9)	31/40 (77.5)	5/40 (12.5)	4/40 (10.0)	
High	12 (23.1)	3/12 (25.0)	4/12 (33.3)	5/12 (41.7)	

^**a**^Determined in paraffin-embedded specimens obtained from 52 patients

**Fig. 8:**
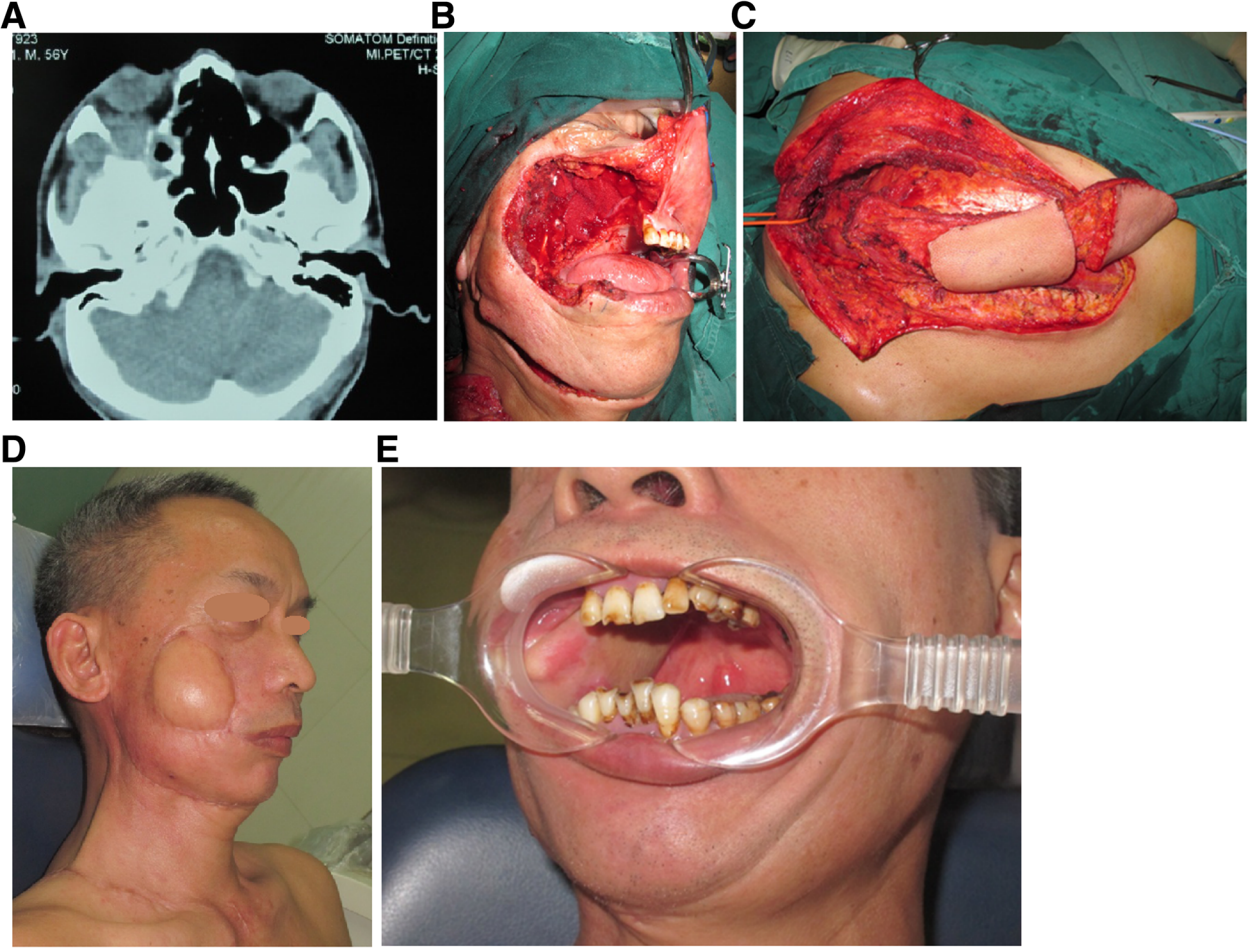
A 56-year-old man with recurrent palatal ACC underwent salvage surgery, including en bloc resection and reconstruction with an extended lower vertical trapezius island myocutaneous flap (TIMF). Computed tomography scan showing that the tumor invaded the middle cranial fossa and posterior cranial fossa (**A**). The patient underwent en bloc resection (**B**), and the extended lower vertical TIMF was harvested (**C**). The folded flap provided an inner and outer lining for repair of the through-and-through defect of the major craniomaxillofacial region (**D**, **E**)

**Fig. 9:**
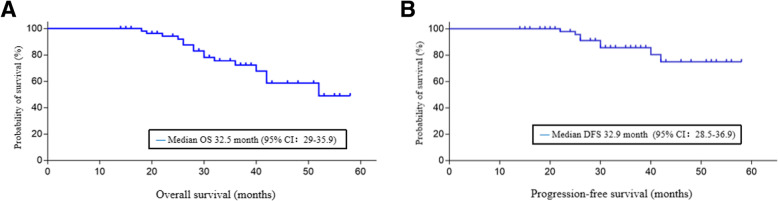
Kaplan–Meier curves of overall survival (**A**) and progression-free survival (**B**) in 58 patients

All patients underwent radical excision with wide safety margins of normal tissues and successful reconstruction of palate defects with a FSAIF or ALTF. No local or general complications developed. The median (range) follow-up duration was 32.9 (14–58) months. Forty-one (71.7%) patients survived without evidence of disease recurrence. Nine patients (15.5%) survived with recurrent tumors (including four with local recurrence [maxilla], three with regional recurrence [skull base] who underwent salvage surgery [Fig. 8], and two with distant metastasis [lungs]); among these patients, five had overlapping recurrence. Eight (13.8%) patients died of distant (two patients with brain metastasis and three with lung metastasis), or multiorgan metastasis (three with metastasis in the lungs and liver) between 22 and 42 months. The median (95% CI) OS was 32.5 (25.0–39.5) months, and the median (95% CI) PFS was 32.9 (28.5–36.9) months (Fig. 9).

Sex, age, tumor site, perineural invasion, bone invasion, and surgical margin status were not associated with survival or recurrence (*P* > 0.05). However, survival and recurrence rates differed according to histopathologic grade (i.e., between the low- and high-grade tumor groups) and TNM stage (i.e., between the stage I–II and stage III–IV disease groups) (*P* < 0.001). In addition, survival and recurrence rates differed according to lymph node metastasis (i.e., between those with and without metastasis), treatment (i.e., between those who received radical excision with versus without radiotherapy), and Ki-67 expression (i.e., between those with low and high expression) (*P* < 0.05). Patient outcomes are summarized in Table 1.

## Discussion

Radical excision with wide safety margins in combination with postoperative radiotherapy is the preferred treatment of ACC in the head and neck region. In this study, all intraoperative proximal margin FS specimens had negative surgical margins, and radical surgical excision was successful. Moreover, adjuvant radiotherapy with a total dose of 60 Gy was administered to the patients with stage III–IV disease. The median follow-up duration was 32.9 months, 71.7% of patients survived without evidence of disease recurrence, 15.5% survived with recurrent tumors, and 13.8% died of regional, distant, or multiorgan metastasis. The median OS was 32.5 months, and the median PFS was 32.9 months. All patients died of local (brain), distant (lung), or multiorgan metastasis (lung and liver).

ACC of the minor salivary glands of the palate is a life-threatening malignant tumor due to its high risk of recurrence and multiorgan metastasis. The characteristic biologic features of ACC include local recurrence, perineural spread, and late distant metastasis. Local control of the disease is difficult because of these features, even in patients with clinically clear surgical resection margins. Our study showed that the cervical lymph node metastasis rate of ACC was very low, and only 5.2% was confirmed. It is suggested that the rationality of selective lymph node dissection needs further study.

Spread via the blood to distant sites, particularly the lungs, usually occurs when the primary tumor has been inadequately treated [15]. In this study, that are more linked to the intrinsic malignancy of the tumor [16]. According to our statistical analysis, the rate of survival without disease was significantly higher in patients with low-grade tumor (90.6%) and stage I–II disease (75.9%) than in patients with high-grade tumor (42.3%) and stage III–IV disease (24.1%). The rates of survival with disease recurrence and death were significantly higher in patients with high-grade tumor (34.6 and 23.1%, respectively) and stage III–IV disease (50.0 and 35.7%) than in patients with low-grade tumor (0 and 6.3%) and stage I–II disease (4.5 and 6.8%) (*P* = 0.0001).

Although radiotherapy is an important adjuvant treatment [17] and we treated 14 patients with stage III–IV disease with surgical excision followed by adjuvant radiotherapy, and the rate of survival without disease was significantly lower among patients who underwent radical excision with radiotherapy (81.8%) than among those who underwent only radical excision (35.7%). Obviously, patients with histopathologic grade I–II tumors or stage I–II disease, but without metastatic tumors, had a better prognosis than those with stage III–IV disease, grade III transformed tumors, or metastatic tumors. In a previous study, patients who received primary treatment with curative intent, mainly surgery, for early stage ACC in the minor salivary glands had a favorable prognosis [18]. Moratin et al. recommend surgical therapy for patients with ACC of the minor salivary glands, including elective neck dissection and microvascular reconstruction, to optimize the planning of adjuvant therapy [19].

Importantly, stage III–IV disease, grade III and transformed tumors, and metastatic tumors indicate advanced disease that warrants aggressive treatment (i.e., local or regional recurrence requires salvage surgery). Early initiation of cyclophosphamide, doxorubicin, and cisplatin chemotherapy may help control metastatic ACC [20]. Chemotherapy can be used to treat patients with brain, lung, or multiorgan metastasis, but such treatment failed in our patients. ACC is an indolent, slow-growing tumor but commonly metastasizes to the lungs and bones. Perineural invasion and bone invasion were noted in 82.8 and 89.7% of patients, respectively; these features can cause local or regional recurrence, hematogenous metastasis, and adverse reactions to radiotherapy or chemotherapy.

Ki-67 expression was significantly higher in the high- than low-grade tumor specimens in this study (*P* < 0.05). Ki-67 may be a subtype-specific marker of ACC in the minor salivary glands of the palate, and a possible prognostic biomarker for tumor progression.

Radical resection with safety margins is the mainstay of treatment for malignant tumors; however, reconstruction of the palate after tumor ablation can be challenging. ACC of the minor salivary glands can be treated radically with surgery, but reconstruction of the defect is rarely reported [14, 21, 22].

The FSAIF has a lower complication incidence when compared to the radial forearm free flap, while maintaining speech and swallowing function [23], and associated with less operative time, shorter hospitalization, fewer perioperative complications, and potentially similar disease recurrence rates compared to free tissue transfer for the reconstruction of oral cavity defects [24]. FSAIF is a reliable and safe method for reconstruction of Brown class II maxillary defects after cancer ablation [25]. However, the best outcomes for Brown class III maxillary defects are achieved with titanium mesh and a free ALTF, which provides good functional and esthetic outcomes after maxillectomy [26].

ACC is a life-threatening malignant tumor owing to its high risk of recurrence; of the rate of tumor-related death within 30 years after primary treatment is high [27]. In this study, the median follow-up of 30 months seems short for a disease like ACC that tends to recur after several years. It is necessary for these patients to continue follow-up.

## Conclusions

We believe that the surgical margins of the specimen must be negative, and the patient may require radiotherapy depending on tumor site, stage, and histologic grade. Radical resection is the best treatment for ACC in the minor salivary glands of the palate. Radical resection and reconstruction with FSAIF is suitable methods for the the treatment of stage I–II ACC of the minor salivary glands of the palate; radical resection and reconstruction with titanium mesh and free ALTF in combination with radiotherapy is an appropriate treatment for stage III–IV tumors. Patients with local or regional recurrence should undergo salvage surgery and adjuvant radiotherapy. Immunohistochemical analysis of Ki-67 expression may provide additional prognostic information.

## Data Availability

Data sharing is not applicable to this article as no datasets were generated or analysed during the current study.
